# Molecular characterization and genetic diversity of *Wolbachia* endosymbionts in bed bugs (Hemiptera; Cimicidae) collected in Paris

**DOI:** 10.1371/journal.pone.0292229

**Published:** 2023-09-28

**Authors:** Dahlia Chebbah, Omar Hamarsheh, Denis Sereno, Nohal Elissa, Sophie Brun, Julie Jan, Arezki Izri, Mohammad Akhoundi

**Affiliations:** 1 Parasitology-Mycology Department, Avicenne Hospital, AP-HP, Bobigny, France; 2 Service Parisien de Santé Environnementale (SPSE), Sous-Direction de la Santé Environnementale et de la Prévention (SDSEP), Direction de la Santé Publique (DSP)-Mairie de Paris, Paris, France; 3 Department of Biological Sciences, Al-Quds University, Jerusalem, Palestine; 4 Institut de Recherche pour le Développement, MIVEGEC, Montpellier, France; 5 Institut de Recherche pour le Développement, InterTryp, Montpellier, France; 6 Agence Régionale de Santé (ARS) Île-de-France, Paris, France; 7 Unité des Virus Émergents (UVE: Aix-Marseille Univ-IRD 190-Inserm 1207-IHU Méditerranée Infection), Marseille, France; University of Bari, ITALY

## Abstract

**Purpose:**

This study aimed to investigate the genetic diversity of *Wolbachia* in field-caught bed bug species in Paris areas.

**Methods:**

The bed bug specimens were captured from various infested localities in Paris and surrounding cities. They belonged to diverse life stages, including egg, nymph, and adult. They were then identified using morphological and molecular approaches. Furthermore, *Wolbachia* was detected, and its genetic diversity was investigated by conventional PCR of 16S-rRNA and *Wolbachia* surface protein (*wsp*) genes.

**Results:**

A total of 256 bed bug specimens belonging to various life stages [adult (183 specimens), nymph (48), and egg (25)] were captured from seven private apartments, five social apartments, three houses, two immigrant residences, and one retirement home situated in 10 districts of Paris and 8 surrounding cities. They were identified as *Cimex lectularius* (237 specimens) and *C*. *hemipterus* (19) using morphological and molecular approaches. The presence and diversity of *Wolbachia* were ascertained by targeting 16S-rRNA and *wsp* genes. Based on molecular analysis, 182 and 148 out of 256 processed specimens were positive by amplifying 16S-rRNA and *wsp* fragments, respectively. The inferred phylogenetic analysis with 16S-rRNA and *wsp* sequences displayed monophyletic *Wolbachia* strains clustering each one in three populations. The median-joining network, including the *Wolbachia* 16S-rRNA and *wsp* sequences of *C*. *lectularius* and *C*. *hemipterous* specimens, indicated a significant genetic differentiation among these populations in Paris areas which was consent with Neighbor-Joining analyses. A phylogenetic analysis of our heterogenic *Wolbachia* sequences with those reported from other arthropod species confirmed their belonging to supergroup F. Moreover, no difference between *Wolbachia* sequences from eggs, nymphs, and adults belonging to the same clade and between *Wolbachia* sequences of *C*. *lectularius* and *C*. *hemipterus* were observed after sequence alignment. Furthermore, no significant correlation was found between multiple geographical locations (or accomodation type) where bed bugs were collected and the genetic diversity of *Wolbachia*.

**Conclusions:**

We highlight a significant heterogeneity within *Wolbachia* symbionts detected in *C*. *lectularius* and *C*. *hemipterus*. No correlation between *Wolbachia* species and bed bug species (*C*. *lectularius* versus *C*. *hemipterus*), physiological stages (egg, nymph, and adult), and sampling location was recorded in this study.

## Introduction

Bed bugs, *Cimex lectularius*, and *C*. *hemipterus* are hematophagous insects feeding almost exclusively on humans. They have a long evolutionary association with humans. They are broadly considered medically important ectoparasites due to the clinical manifestations of bite reactions and the psychological effects elicited by their infestation [1,2]. Although they are not regarded as biological vectors of human pathogenic agents, over 45 pathogens, including bacteria, viruses, fungi, and parasites, have been detected in bed bugs [1,3]. Since the last three decades, bed bugs have experienced a significant re-emergence, attributed mainly to the increase in travel and their resistance to insecticides [4]. Bed bug infestations have become common in private or public settings, including hotels, elder residences, hospitals, and public transportation.

Like many insects, bed bugs depend on their bacterial symbionts to get essential nutrients and extend their adaptive potential to deal with challenging environments [5].

*Wolbachia*, a Gram-negative alpha-proteobacterium, is the most prevalent endosymbiotic bacterial group, associated with over 60% of insect species [6,7]. These intracellular bacteria are transmitted vertically and are essential for various host reproductive processes such as cytoplasmic incompatibility, feminization, male death, parthenogenesis, increased or decreased fitness, and obligate symbiosis [8–11]. Elimination of *Wolbachia* symbionts result in retarded growth and sterility of the insect host. They express a high genetic diversity, likely due to their interactions with many invertebrate hosts [12,13]. They are classified into 18 monophyletic lineage groups from A to T in various arthropods and filarial nematodes. *Wolbachia* belonging to supergroups C, D, and J are exclusively found in filarial nematodes (Onchocercidae) [14–16]. Supergroup L contains exclusively plant-parasitic nematodes (Pratylenchidae) [17,18]. Supergroup F is a common supergroup for insects and filarioids [19]. The latter (T) is a new supergroup recently identified in *C*. *hemipterus* from Senegal [20].

The bed bugs’ need for regular blood feeding on humans and their residence near the host has resulted in an increased potential for transmission of pathogens, including arboviruses. Fort Morgan virus (FMV), Tonate virus (TONV), and Kaeng Khoi (KKV) viruses (two first viruses belonging to the *Alphavirus* genus and the latter belonging to *Orthobunyavirus*) are such arboviruses reported from swallow bugs (*Oeciacus vicarius*) and bat bugs (*Cimex insuetus* and *Stricticimex parvus*) [21–23]. Despite limited knowledge on the pathogenicity of FMV and KKV viruses, TONV can cause fatal encephalitis in humans [24]. *Wolbachia* can affect the Arboviral life cycle by supporting host factors and strengthening host antiviral mechanisms [25,26]. Although the interaction of *Wolbachia*-arbovirus is less known in bed bugs, accurate species-specific *Wolbachia* determination is essential, which may help control bed bug populations. Conversely, *Wolbachia* transmission mode may affect symbiont-host phylogenetic co-variation [27]. The increasing number of *Wolbachia* sequences allows a detailed characterization of their genetic diversity thanks to significant progress in molecular methods over the last three decades.

Despite knowledge of the prevalence of *Wolbachia* within a wide range of arthropod hosts, including bed bugs, only a few studies analyze the *Wolbachia* genetic diversity of human bed bug species. This study concentrated on *Wolbachia* species composition and genetic diversity in bed bugs collected in various districts of Paris areas.

## Materials and methods

The Parasitology Department of Avicenne hospital (Bobigny, France) is a reference center for ectoparasitic diseases in Paris. The present study was conducted between April and June 2021 on the patients referred for suspected bed bug infestation. Participation in this study was voluntarily based on the participants’ decision. Before bed bug collection, the participants were convinced about this study’s goal and verbally agreed to bed bug collection. Moreover, absolute privacy was assured to inhabitants of inspected locations.

### Bed bug collection

Bed bug samples were collected alive using a handheld vacuum cleaner (Dyson V7 trigger) and entomological forceps from various infested locations in Paris areas. They included private houses, apartments, social complexes, immigrant residences, and retirement home. The samples were transferred to the laboratory in plastic pots with folded papers. The gathered samples were then identified under a stereo microscope (Olympus SZ61, Yokohama, Japan) according to the identification keys of Usinger [28] and Walpole [29]. All the specimens were labeled and kept at -20°C for further molecular analysis.

### DNA extraction, PCR amplification, and species assignation

The caught bed bugs’ DNA was extracted individually using Chelex 10% (Bio-Rad, CA, USA). The processed specimens were undergone for species identification by conventional PCR targeting the cytochrome oxidase 1 (COI) gene [30].

The extracted specimens were then subjected to conventional PCR amplifications targeting two genes to assess the genetic diversity of *Wolbachia* symbionts within and between bed bug populations. The first PCR batch was performed by amplification of 1500 bp of 16S-rRNA using forward (16suF: 5′-GAGTTTGATCCTGGCTCAG-3′) and reverse (16suR: 5′-GTTACCTTGTTACGACTT-3′) primers [12]. The PCR reactions were performed under the following conditions: initial denaturation at 94°C for 1 min, followed by 35 cycles of 95°C for 30 s, annealing at 57.5°C for 40 s, 72°C for 30 s, and a final extension at 72°C for 8 min. The second PCR of 600 bp fragments of the *Wolbachia* surface protein (*wsp*) gene was performed using forward (81F: 5′-TGGTCCAATAAGTGATGAAGAAAC-3′) and reverse (wsp691R: 5′-AAAAATTAAACGCTACTCCA-3′) primers [13]. The PCR program was carried out with an initial denaturation at 95°C for 5 min, followed by 35 cycles of 94°C for 40s, 45°C for 40s, 72°C for 1 min, with a final extension of 72°C for 5 min. Negative and positive controls were included in each PCR batch. The amplicons were analyzed using electrophoresis on 1.5% agarose gel containing ethidium bromide.

### Genetic diversity analysis and phylogenetic reconstruction

PCR products were then subjected to bidirectional DNA sequencing with the same primer pairs used for amplification. The sequences were edited, aligned, and compared with homologous sequences from GenBank using the BLAST (Basic Local Alignment Search Tool) (www.ncbi.nlm.nih.gov/BLAST). Strains were assigned to species level, based on ≥99% homology to GenBank sequences. All nucleotide sequences of *Wolbachia* were deposited in GenBank with the assigned accession numbers NK65843901 to NK65844051. Sequence alignment was performed with the BioEdit v7.0.0 software [31], and the phylogenetic analysis was carried out using MEGA v.6 software [32]. The inferred phylogenetic trees of *Wolbachia* species (identified in this study) and homonym sequences from GenBank were constructed using the neighbor-joining (NJ) method and bootstrap values determined by 1000 replicates. To display the genetic relationships within *Wolbachia* populations, the median-joining algorithms were implemented using NETWORK v.5 software [33].

## Results

A total of 256 bed bug specimens were captured from various human dwellings. The latter includes seven private apartments, five social apartments, three houses, two immigrant residences, and one retirement home situated in 10 districts of Paris and eight surrounding cities. The gathered bed bugs belonged to diverse life stages, including adults (183 specimens), nymphs (48), and eggs (25). The highest sample number was from the 19^th^ district of Paris (41 specimens), followed by the 18^th^ (40), and the lowest number of specimens was collected in the Neuiy-Sur-Seine district (2). Morphological identification of adult specimens disclosed the presence of 2 species, *C*. *lectularius* (237 specimens) and *C*. *hemipterus* (19). Immature life stages and adult specimens underwent molecular identification using conventional PCR targeting the mitochondrial COI gene [30]. BLAST analysis of COI gene sequences revealed ≥98% identity with counterpart sequences of *C*. *lectularius* (e.g., KR035952) and *C*. *hemipterus* (e.g., MN915235) in GenBank. Information on the bed bugs’ origin, species composition, and their positivity rate with *Wolbachia* sp are given in **Table 1**.

**Table 1:** Geographical dispersion of *Wolbachia* sp. identified in field-caught bed bug specimens in Paris and surrounding cities.

District	Accommodation type	Bed bug species	Life stage (number of specimens)	Number of positive specimens		Positivity rate (%)
				16S-rRNA	*wsp*	
Paris 14^th^	Private apartment	*C*. *lectularius*	Adult (8)	6	5	75
Paris 19^th^	Immigrant residence	*C*. *lectularius*	Adult (26), nymph (12) and egg (3)	38	34	92.7
Paris 20^th^	Social apartment	*C*. *lectularius*	Adult (7)	5	3	71.4
Paris 11^th^	Private apartment	*C*. *lectularius*	Adult (5)	4	3	80
Paris 12^th^	Private apartment	*C*. *hemipterus*	Adult (8)	6	4	75
Paris 7^th^	House	*C*. *lectularius*	Adult (11) and egg (6)	13	10	76.4
Paris 17^th^	Private apartment	*C*. *lectularius*	Adult (11)	9	6	81.8
Paris 10^th^	Private apartment	*C*. *lectularius*	Adult (6)	5	4	83.3
Paris 18^th^	Social apartment	*C*. *lectularius*	Adult (21), nymph (11), and egg (8)	32	29	80
Paris 13^th^	Private apartment	*C*. *hemipterus*	Adult (6)	4	3	66.6
Villjuif	Retirement home	*C*. *hemipterus*	Adult (5)	2	2	40
Montreuil	Private apartment	*C*. *lectularius*	Adult (5)	3	2	60
Saint-Denis	Immigrant residence	*C*. *lectularius*	Adult (15) and nymph (7)	9	7	40.9
La Courneuve	Social apartment	*C*. *lectularius*	Adult (12) and egg (3)	11	8	73.3
Stains	Social apartment	*C*. *lectularius*	Adult (10) and nymph (7)	12	9	70.5
Neuiy -Sur-Seine	House	*C*. *lectularius*	Adult (2)	2	2	100
Sevran	House	*C*. *lectularius*	Adult (9) and nymph (4)	10	8	76.9
Bobigny	Social apartment	*C*. *lectularius*	Adult (16), nymph (7) and egg (5)	11	9	39.2

**Fig 1:**
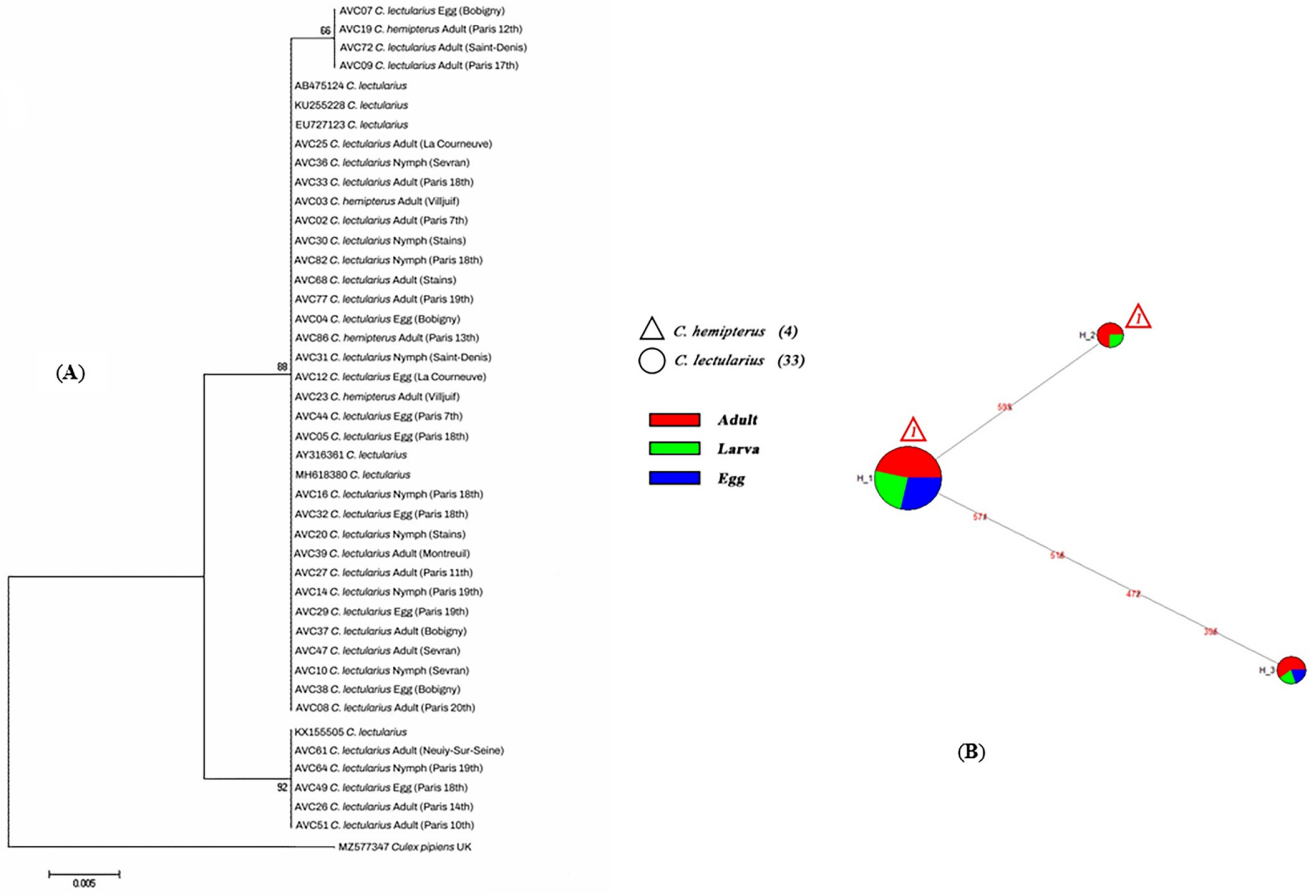
**A**) Neighbor-joining (NJ) tree reconstructed from the 16S-rRNA sequences of *Wolbachia* isolates detected in various life stages (egg, larva & adult) of bed bug specimens we collected (beginning with AVC) and sequences from GenBank, **B**) Median-joining network for the 16S-rRNA sequences of *C*. *lectularius* and *C*. *hemipterus* specimens processed in this study.

**Fig 2:**
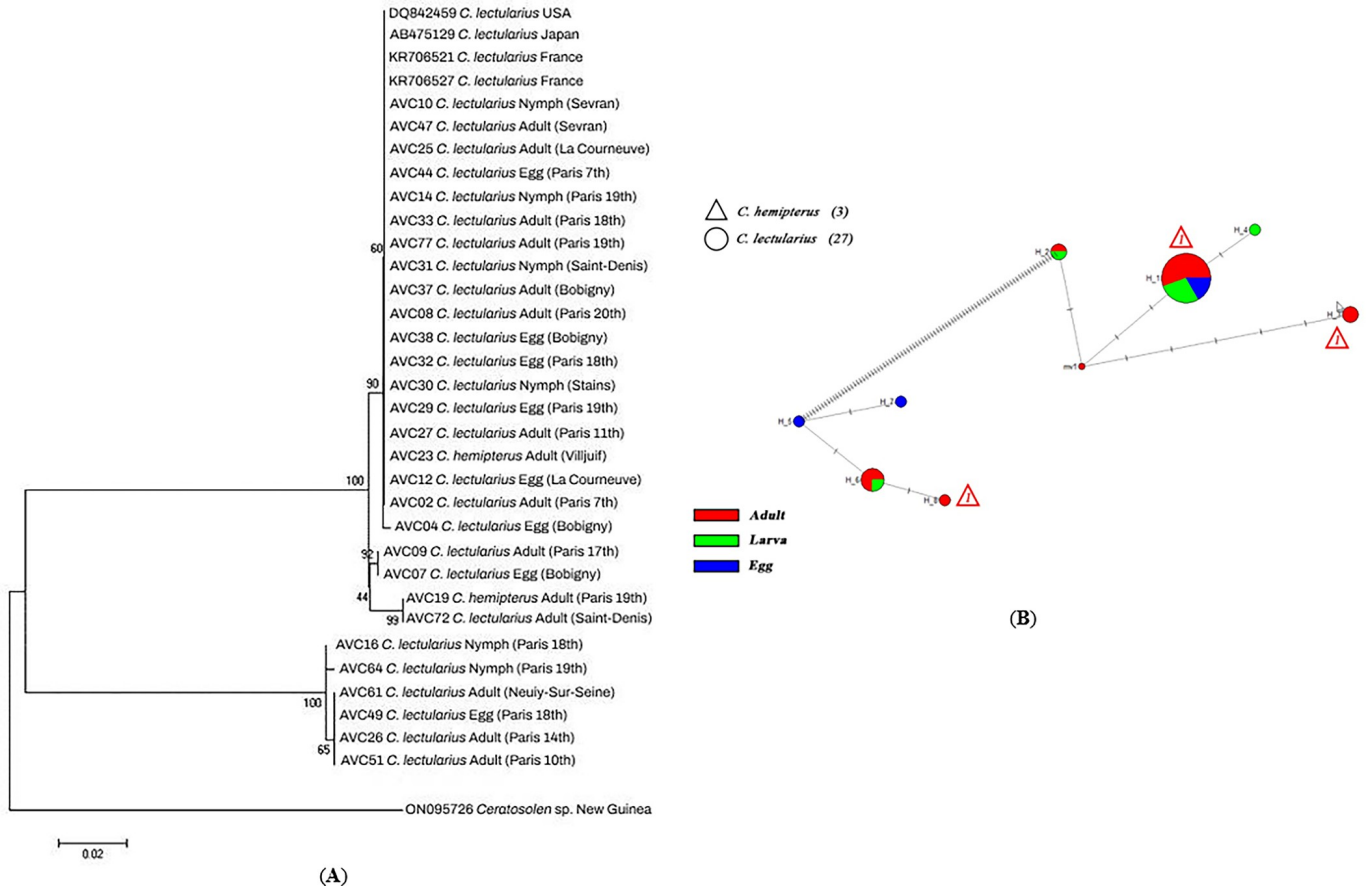
**A**) Neighbor-joining (NJ) tree reconstructed from the *wsp* sequences of *Wolbachia* isolates detected in various life stages (egg, larva & adult) of bed bug specimens we collected (beginning with AVC) and sequences from GenBank, **B**) Median-joining network analysis of *wsp* sequences for the same specimens.

Based on molecular analysis, 182 and 148 out of 256 bed bug specimens collected were positive for *Wolbachia* sp. revealed by amplifying the 16S-rRNA and *wsp* fragments, respectively. The acquired sequences were identified at the species level,based on identity equal to or more than 99% compared with homologous 16S-rRNA (e.g., AY316361, OP730740) and *wsp* (e.g., KR706527, QXIM01000003) sequences collected in GenBank. The inferred NJ phylogenetic trees constructed by 16S-rRNA and *wsp* sequences using the p-distance substitution model and supported by bootstrap displayed the presence of three monophyletic populations for each analysis separately (**Figs 1A and 2A**).

Furthermore, the clustering displayed by the median-joining network agreed well with the topology of the phylogenetic trees generated by NJ analysis. The median-joining network, including the *Wolbachia* sequences of *C*. *lectularius* and *C*. *hemipterous* specimens processed in the present study, indicated a significant genetic differentiation among these populations in Paris areas with three populations for 16S-rRNA and *wsp* (**Figs 1B and 2B**).

In addition, the genetic distance of *Wolbachia* 16S-rRNA (SI.1A) and *wsp* (SI.1B) is given in **S1 File**.

**Fig 3:**
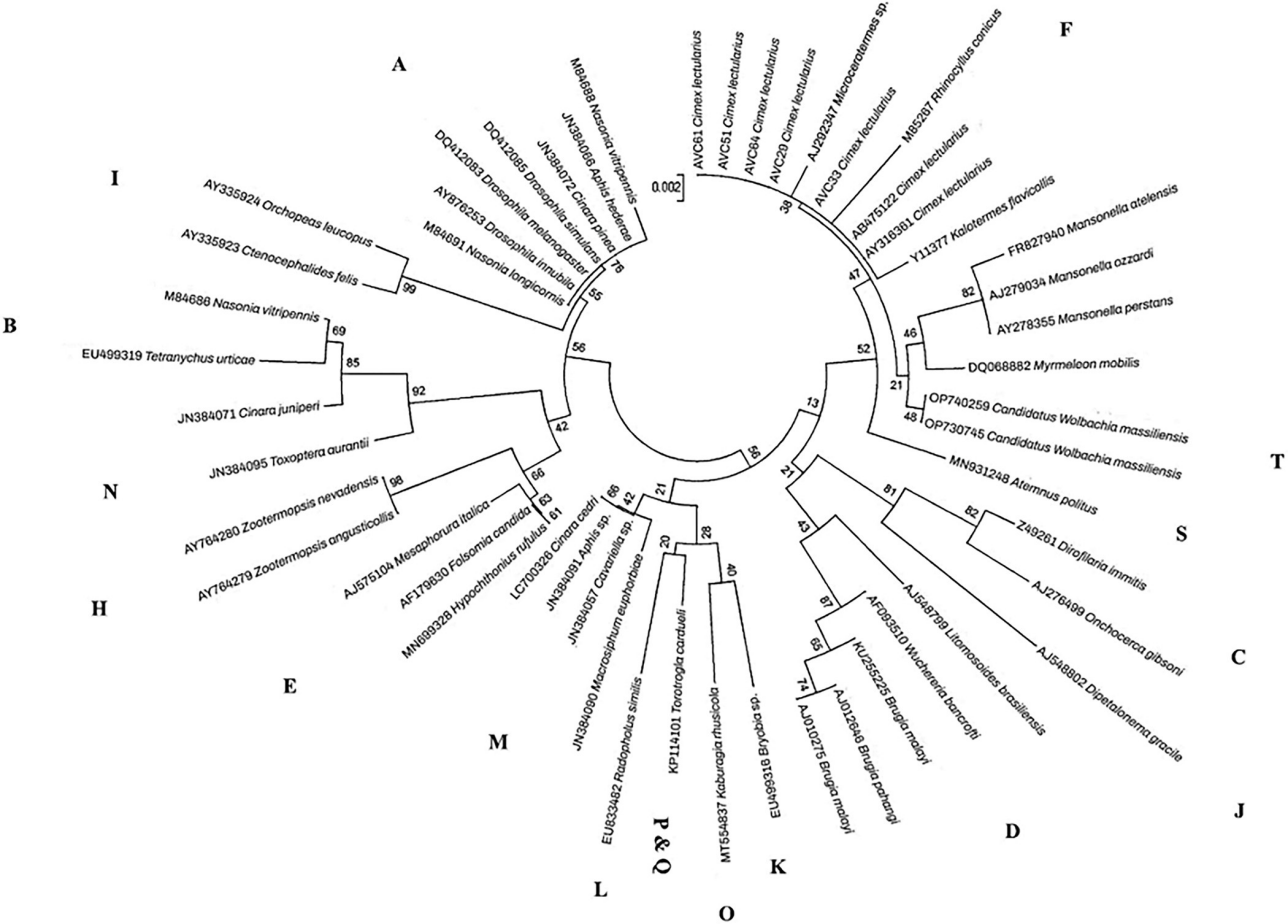
Unrooted phylogenetic tree of *Wolbachia* 16S sequences belonging to specimens we collected (named AVC) and *Wolbachia* strains reported from diverse arthropod and helminth hosts collected from GenBank.

Further unrooted phylogenetic analysis of 16S-rRNA sequences of *Wolbachia* sp. identified in this study and homologous counterparts of other arthropods and nematodes collected in GenBank confirmed that all our *Wolbachia* specimens belonged to supergroup F (**Fig 3**).

**Fig 4:**
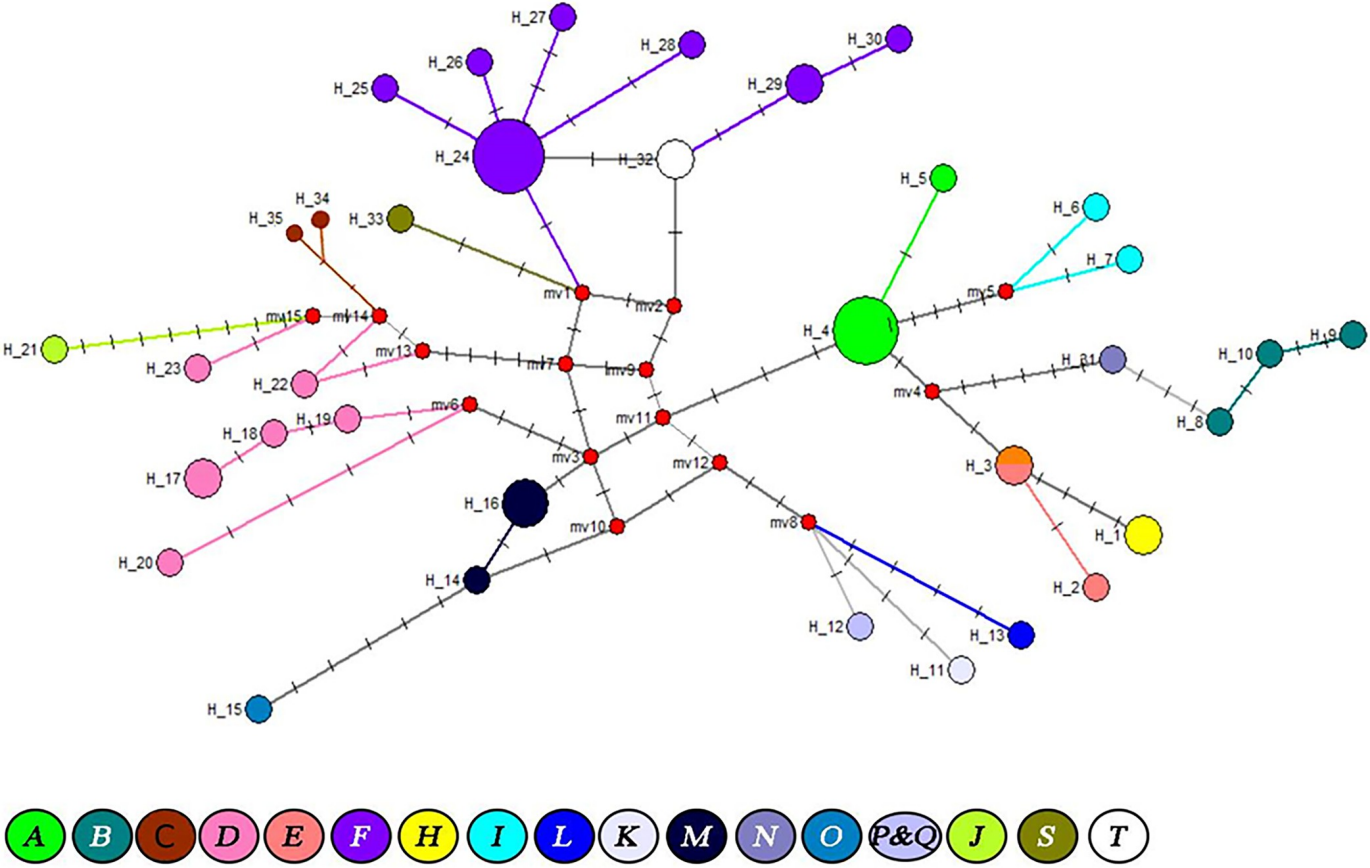
Global network analysis of *Wolbachia* 16S-rRNA sequences of bed bug specimens analyzed in this study and homologous counterparts of other arthropods and nematodes collected in GenBank.

High genetic diversity within and between *Wolbachia* taxa associated with diverse arthropod and nematode species was depicted by global network analysis of the 16S-rRNA sequences (**Fig 4**).

The distributions of 16S-rRNA and *wsp* haplotypes within *Wolbachia* in Paris areas processed in the present study are shown in **S2 File**.

## Discussion

Bed bugs are regarded as one of the most critical human ectoparasites with a global geographical distribution; the investigation of *Wolbachia*, a symbiont of bed bugs, as a non-chemical biological method in controlling bed bugs has received significant attention in recent years. They were the subject of some investigations indicating the high prevalence of this symbiont in various bed bug populations worldwide [34,35]. Nevertheless, the genetic diversity of these symbionts, which may impact their application in bed bug control, has not been thoroughly addressed. One of the few studies on *Wolbachia*’s presence in bed bugs was conducted by Hypsa and Aksoy [8], in which two different inherited bacterial symbionts from the ovary tissue of *C*. *lectularius* were characterized by targeting the 16S-rRNA gene. They included *Wolbachia* and BEV (bacterial endosymbiont of the leafhopper, *Euscelidius variegatus*). A phylogenetic study of *Wolbachia* symbionts in *Oeciacus vicarious* (cliff swallow bug) and *C*. *lectularius* targeting 16S-rRNA and *FtsZ* genes revealed a monophyletic group belonging to the clade F [34]. In another investigation performed on the *C*. *lectularius* populations of five North American regions (California, Connecticut, Florida, New York, and Toronto, Canada) and one African region (Macha, Zambia), a *Wolbachia* prevalence of 83–100% with no significant difference in *Wolbachia* frequency between geographic regions, between sexes, or between life stages (adult versus nymph) was reported [36]. Determination of the whole genome of *Wolbachia* in *C*. *lectularius* (wCle) indicated its similarity to the genomes of insect-associated facultative *Wolbachia* strains [10]. A recent study on the *C*. *hemipterus* of Senegal highlighted the diversity of *Wolbachia* genotypes and presented *W*. *massiliensis* as a type strain of newly discovered supergroup T [20]. In this study, a high level of *Wolbachia* prevalence (71.1% of processed specimens) was detected, which was significantly more than what we reported in our previous article (~38%) from other regions of France [37]. This is the first report of *Wolbachia* prevalence in Paris areas’ bed bug populations and the second one, besides our previous article, in France. No difference between *Wolbachia* sequences from eggs, nymphs, and adults belonging to the same clade was observed after sequence alignment using BioEdit. This can be explained by the fact that *Wolbachia* is a maternally inherited bacterium with no significant genetic difference between *Wolbachia* of a female bed bug and its descendants [38,39]. In addition, no difference between *Wolbachia* sequences of *C*. *lectularius* and *C*. *hemipterus* was recognized. Despite the genetic diversity of *Wolbachia* strains identified in various bed bug populations of infested locations, no significant correlation was found between multiple geographical locations where bed bugs were collected and the genetic diversity of *Wolbachia*. Furthermore, high heterogeneity of *Wolbachia* 16S-rRNA and *wsp* sequences was observed regrouped in three populations with no hybrid or genetic recombination. These findings are consent with investigations performed by Schuler et al. [40] and Webb et al. [41] on the *Belonocnema treatae* (gall wasp) and *Polyrhachis* (spiny ants), in which no strict association between *Wolbachia* diversity and geography of studied insect hosts was reported. Supergroup F is a phylogenetic clade composed of some arthropod and filarial-infecting strains of *Wolbachia* [42,43]. Supergroup T, characterized recently as a new *Wolbachia* strain associated with *C*. *hemipterus* from Senegal, is closely related to supergroups F and S of insects and supergroup D of filarial nematodes (Fig 3) [20]. A global phylogenetic analysis of our 16S-rRNA sequences and *Wolbachia* sequences reported from diverse nematodes and arthropods collected from GenBank demonstrated that high heterogenic *Wolbachia* sequences detected in this study belonged to supergroup F (Figs 3 and 4). These findings highlight the genetic diversity of bed bugs *Wolbachia* symbionts in the framework of the *Wolbachia* strains belonging to 18 supergroups reported from other arthropods and nematodes. There is growing evidence that various *Wolbachia* strains carry blocking effects in diverse arthropod species [44]. The genetic diversity of *Wolbachia* may therefore have potential application in biologic control of bed bugs and the pathogenic agents reported to harbor by them. In the recent years, pest control programs are developing that rely on the driving ability of artificial *Wolbachia* infections that reduce pathogen transmission [45,46]. A highly enough release of insects’ infected individuals beyond threshold infection frequencies is reported to be successful in population replacement [45].

## Conclusions

We highlight *Wolbachia* prevalence in 71.7% of processed specimens belonging to various life stages (eggs, larva and adult) and bed bug species (*C*. *lectularius* and *C*. *hemipterous*). No significant correlation was found between various geographical locations or life stages and genetic diversity of *Wolbachia*. The phylogenetic reconstruction using 16S-rRNA and *wsp* sequences demonstrated high heterogeneity and the occurrence of three monophyletic populations. Further global analysis of 16S-rRNA revealed that these sequences belonged to the supergroup F of *Wolbachia*. These findings complete our knowledge of the *Wolbachia* symbionts in bed bugs and their competency in the biological control of these ectoparasites.

## Supporting information

S1 File The pairwise genetic distances matrix of *Wolbachia* 16S-rRNA and *wsp* sequences. (ZIP)

S2 File Haplotype diversity within 16S-rRNA and *wsp* fragments of *Wolbachia* in bed bug specimens. (ZIP)
